# A new species of shrimp of the genus *Anachlorocurtis* Hayashi, 1975 from the Red Sea, with range extension of *A. commensalis* Hayashi, 1975 (Crustacea, Decapoda, Pandalidae)

**DOI:** 10.3897/zookeys.407.7457

**Published:** 2014-05-08

**Authors:** Ivona Horká, Sammy De Grave, Zdeněk Ďuriš

**Affiliations:** 1 University of Ostrava, Faculty of Science, Department of Biology and Ecology, Chittussiho 10, CZ-71000 Ostrava, Czech Republic; 2 Charles University in Prague, Faculty of Science, Department of Ecology, Viničná 7, CZ-12844 Prague, Czech Republic; 3 Oxford University Museum of Natural History, Parks Road, Oxford, OX1 3PW, United Kingdom

**Keywords:** Caridea, Pandalidae, *Anachlorocurtis occidentalis*, new species, *Antipathes*, Antipatharia, Red Sea

## Abstract

A new species of pandalid shrimp *Anachlorocurtis occidentalis* **sp. n.**, associated with antipatharian corals, is described and illustrated from the north-eastern Red Sea. This new species is closely related to *Anachlorocurtis commensalis* Hayashi, 1975, the only other species in the genus, and can be distinguished by the more slender body and appendages; the carapace with 3 large, and one small, subtriangular lobes in the middorsal line; a flattened dorsal outline of the third abdominal segment; the sixth abdominal segment twice as long as fifth one; propodi of the ambulatory pereiopods bearing only a single posterior spinule; and harbouring 3–5 pairs of dorsolateral spines on the telson. A revised generic diagnosis is provided here to accommodate the present new species. The genetic divergence of mitochondrial gene cytochrome c oxidase subunit I (COI) between *Anachlorocurtis occidentalis* sp. n., and *A. commensalis* is 15.2–15.4%. Molecular analysis also confirmed a sister position of the genus *Anachlorocurtis* to *Miropandalus*. The present records of *A. commensalis* from Taiwan constitute an extension of the known range of the species.

## Introduction

Shrimps of the family Pandalidae include 23 genera and 190 species (De Grave and Fransen 2011, Komai and Tsuchida 2014), with their greatest diversity occurring in boreal to temperate Atlantic and Pacific waters, especially in low-latitudinal areas of the Indo-West Pacific, and with relatively few species at similar latitudes in the southern hemisphere. In tropical regions, numerous pandalid species are known, but largely restricted to deeper, colder water (Bauer 2004). Overall, this is a diverse group of medium to large-sized, mainly epibenthic shrimps with a typical natant body form possessing well-developed rostra and long slender or robust legs (Bruce 1983, Bauer 2004). With almost no exception, species of Pandalidae are free-living and the association with other invertebrates is restricted to three species: *Chlorotocella gracilis* Balss, 1914, *Anachlorocurtis commensalis* Hayashi, 1975, and *Miropandalus hardingi* Bruce, 1983. *Chlorotocella gracilis* has been reported in association with a jellyfish and gorgonarian corals (Hayashi and Miyake 1968, Li and Davie 2006, Hayashi 2007), although those associations are perhaps best regarded as accidental, as numerous reports exist on free-living specimens (Bruce 1972). The other two species are obligate symbionts of antipatharian black corals (Anthozoa, Antipatharia) *Antipathes* spp., *Myriopathes japonica* (Brook, 1889) and *Cirrhipathes* spp. (Hayashi 1975, Bruce 1983, 1991).

The genus *Anachlorocurtis* Hayashi, 1975 has, up to now, only included a single species, *Anachlorocurtis commensalis* known from Japan (Hayashi 1975, 2007) and recorded herein from Taiwan. The new species *Anachlorocurtis occidentalis* sp. n. was found in the Red Sea, thus considerably extending the genus’ geographical range, equally associated with antipatharian corals. The external morphology of both *Anachlorocurtis* and *Miropandalus* are unique, with their body being well adapted to life with their hosts. They are small, slender, and compressed animals with a cryptic colour pattern (Kawamoto and Okuno 2003, Minemizu 1996, 2013), short (*Anachlorocurtis*), or fully reduced (*Miropandalus*) rostra, specialised mouth-parts, and comparatively large and few eggs (Hayashi 1975, Bruce 1983, 1991).

The following abbreviations are used: COI, mitochondrial gene cytochrome c oxidase subunit I; fcn, field collection numbers for Aqaba (Aq), Eilat (ISR) and Taiwan (Tw, TAI); SEM, scanning electron microscope; PoCL, post orbital carapace length; spm(s), specimen(s); RL, rostrum length (measured from the posterior orbital margin to the distal edge of the most anteriorly advanced tooth of the rostrum); TL, total length of the body (measured from tip of the rostrum to the distal end of the telson, posterior telson spines not included); MSS, Marine Science Station, Aqaba, Jordan; NTOU, National Taiwan Ocean University; OUMNH.ZC, Zoological Collection, Oxford University Museum of Natural History, Oxford; RMNH, Naturalis Biodiversity Center, Leiden; UO, University of Ostrava.

## Material and methods

Specimens of *Anachlorocurtis occidentalis* sp. n. were collected in the Red Sea (Aqaba, Jordan, in 2008 and 2009; Eilat, Israel, in 2011), and *Anachlorocurtis commensalis* in the South China Sea (southern Taiwan, in 2009, 2011, 2012), respectively, from antipatharian corals using hand nets and SCUBA equipment. Samples were preserved in 80% ethanol for morphological studies and in 96–99% ethanol for molecular analysis.

The terminal segments of the first and second pereiopods of both species examined were photographed under SEM. One specimen of each species (*Anachlorocurtis occidentalis* sp. n. UO Aq09-30A and *Anachlorocurtis commensalis* UO Tw12-79) was dehydrated by a series of acetone/ethanol solutions of concentrations 30, 50, 70, 80, 90, 95 and 100% acetone in incremental 30 minute steps. Acetone was removed by the CO_2_ critical point method (POLARON E3000 Critical Point Drying Apparatus) with dried specimens gold-coated (Automatic Sputter Coater: JEOL JFC-1300) and examined under a scanning electron microscope (SEM-JEOL JSM-6610LV).

Total genomic DNA was extracted from abdominal muscle tissue or eggs using the DNeasy Blood & Tissue isolation Kit (QIAGEN) according to the manufacturer’s instructions. For amplifying the segment of mitochondrial protein-coding gene COI, with a polymerase chain reaction, the universal pair of primers LCO1490/HCO2198 was used (Folmer et al. 1994). The PCR was conducted in 25–30 μl reaction volume containing: 2–3 μl DNA template, 0.3 μM each primer, 0.15 mM dNTP, 0.7 units of *Taq* polymerase, distilled water, 10 × PCR buffer and 2.5 mM of MgCl_2_. PCR cycling profile was as follows: 2.5 min at 94 °C for initial denaturation, followed by 40 cycles of 30 s at 90 °C, 1 min at 48 °C, 1 min at 72 °C and the final extension step at 72 °C for 10 min. PCR products were purified using GenElute PCR clean-up kit (Sigma). Sequencing reactions were carried out using the ABI3730XL DNA Sequencer at Macrogen, Inc. Sequences obtained were deposited in GenBank (Table 1), from which additional sequences were used. Sequences were aligned using MUSCLE (Edgar 2004). The divergence of the analysed mitochondrial gene between both species was detected using the Kimura 2-parameter model. A further seven pandalid species (including *Miropandalus*, a morphologically closely related genus) and one stenopod shrimp (outgroup) were included in the phylogenetic analysis. The best-fit nucleotide substitution model under Bayesian Information Criterion (GTR+G+I, General Time Reversible) was selected. Phylogenetic reconstruction of COI data set was performed in a maximum likelihood (ML), bootstrap was done with 1,000 replicates. All analyses were conducted using MEGA v5.2.1 (Tamura et al. 2011).

**Table 1.** Species used in the molecular analysis. Sampling location is given, as is GenBank accession numbers (COI), and voucher identification numbers of specimens examined. (ii, v) – see Material; * - Sequences obtained from GenBank.

Taxa	Sampling location	GenBank accession #	Voucher ID
**Pandalidae**			
*Anachlorocurtis occidentalis* sp. n. (ii)	Jordan, Aqaba	KJ690257	RMNH.CRUS.D.56174
*Anachlorocurtis occidentalis* sp. n. (v)	Jordan, Aqaba	KJ690256	RMNH.CRUS.D.56177
*Anachlorocurtis commensalis* Hayashi, 1975	Taiwan, Nanvan	KJ690258	RMNH.CRUS.D.56182
*Miropandalus hardingi* Bruce, 1983	Taiwan, Nanvan	KJ690259	UO Tw11-20A
*Heterocarpus ensifer* A. Milne-Edwards, 1881	Guadeloupe	*AY612858	NTOU
*Heterocarpus gibbosus* Spence Bate, 1888	Philippines, Panglao	*GQ302742	NTOUM00797
*Pandalus borealis* Krøyer, 1838	Canada, Quebec	*FJ581839	PB01CN0406
*Pandalus montagui* Leach, 1814	Canada, New Brunswick	*FJ581840	GSL31-52
**Stenopodidae (outgroup)**			
*Stenopus hispidus* (Olivier, 1811)	Vietnam, Nhatrang Bay	KJ690260	UO V10-17

## Systematics

### Superfamily Pandaloidea Haworth, 1825
Family Pandalidae Haworth, 1825

#### Genus Anachlorocurtis

Hayashi, 1975

http://species-id.net/wiki/Anachlorocurtis

##### Generic diagnosis

(modified from Hayashi 1975). Small-sized shrimps. Rostrum short, deep, not reaching end of eyes; anterior and dorsal margins with 2–6 small teeth in adult females; rostrum styliform, anteriad in adult males. Carapace with 2 large compressed dorsal lobes, with small epigastric tooth and one small lobe near posterior margin; anterior (i.e., postrostral) lobe just behind orbit, similar and subequal to rostrum, with 2–4 small teeth anteriorly and small epigastric lobe posteriorly; large posterior, anteriorly hooked lobe on distal third of dorsal midline; antennal tooth marginal, acute; supraorbital, hepatic and pterygostomial teeth absent. Abdomen smooth, without spines or spiniform processes, sixth segment elongate, with posterior lobes obtuse. Telson with 3–5 pairs of small dorsal spines on lateral margins; posterior margin rounded with 5 pairs of spines. Eyes well developed, cornea with apical tubercle, accessory pigment spot lacking, stalk slightly longer than corneal length. Antennular peduncle elongate; basal segment with deep medioventral keel; stylocerite short, anteriorly truncate with produced distolateral tooth and medial angle pointed; distal 2 peduncular segments short; upper and lower flagella uniramous, short. Scaphocerite well developed, distolateral spine not exceeding lamella. Mandible without palp. Maxillula with palp bearing 2 long setae, upper lacinia broader that lower lacinia. Maxilla with bi-setose palp, with simple, broad, distal endite and bilobed proximal endite, scaphognathite well developed, posterior lobe not particulary elongate. First maxilliped with large palp; exopod without flagellum but with elongate setose caridean lobe; endites feebly separated; podobranch lacking. Third maxilliped long, slender, with elongate lateral lobe; merus, basis and ischium (i.e., antepenultimate segment) fused; exopod, epipod and arthrobranch absent. Pleurobranchs present above all pereiopods. First pereiopods slender, not chelate, with dactylus reduced; ischium without lamellar expansion. Second pereiopods slender, chelate, equal with small subspatulate fingers bearing irregularly denticulate cutting edges; carpus three-segmented. Ambulatory pereiopods with dactylus slender, simple; propodus with 1–2 spinules on ventral margin, distoventral spinules lacking or present, small; pereiopod 3–5 meral spinulation 2-2-(0-2), respectively. Endopod of first pleopod reduced to small setose lobe far overreached by functional appendix interna in males, and more reduced, without cincinnuli, in females. Uropod with elongate branches.

##### Generic distribution.

Kii Peninsula to Ruykyus, Japan, at depths of 8–15 m (Hayashi 1975, Kawamoto and Okuno 2003); southern Taiwan, depths 10–27 m (this report), and northeastern Red Sea, depths 4–55 m (this report).

#### Anachlorocurtis occidentalis sp. n.

http://zoobank.org/AA95EADC-5095-4590-A5D5-16B902C88249

http://species-id.net/wiki/Anachlorocurtis_occidentalis

**Figure 1.**
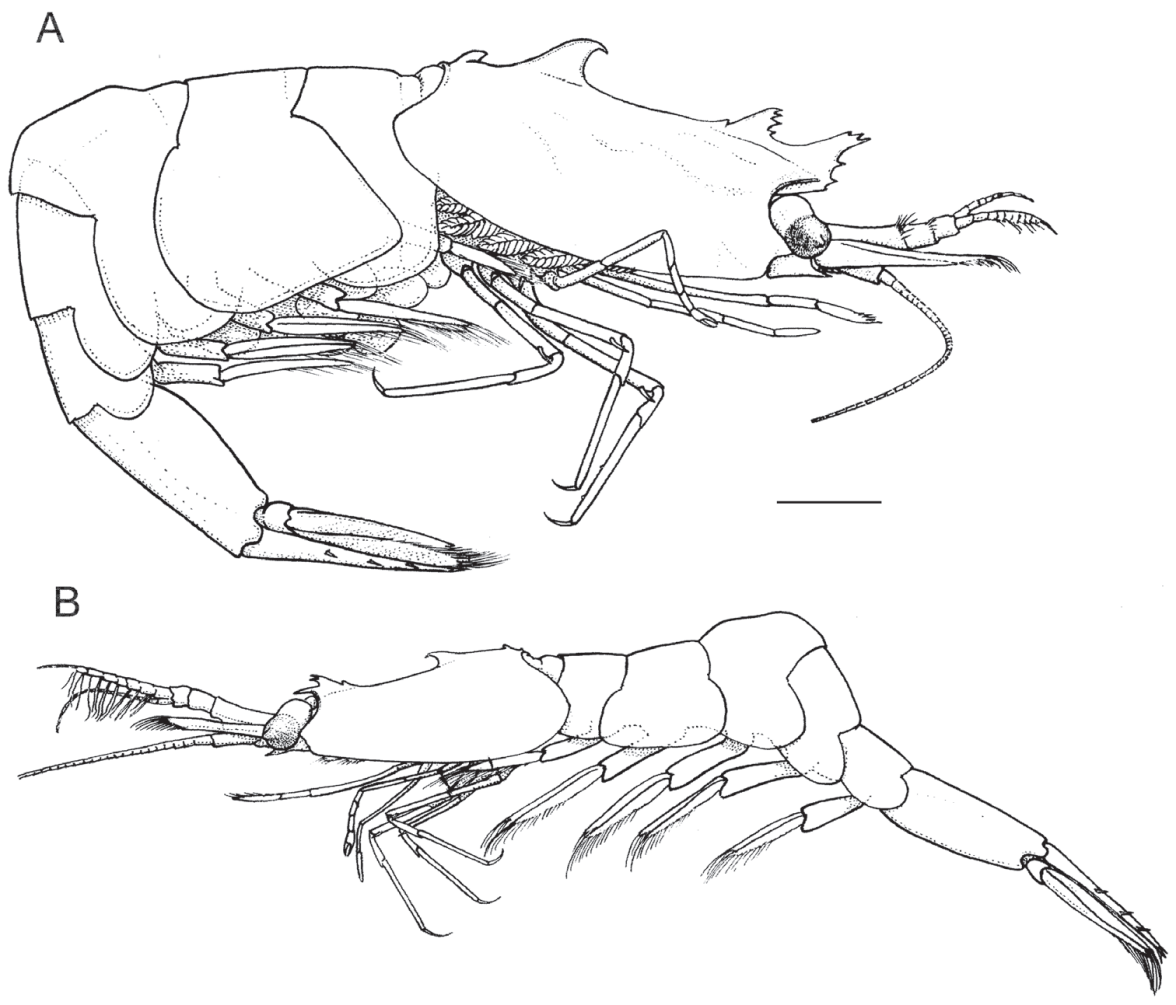
*Anachlorocurtis occidentalis* sp. n., total aspect. **A** holotype, ovigerous female (RMNH.CRUS.D.56175) **B** allotype, male (RMNH.CRUS.D.56176). Scale bar equals 1 mm.

Figs 1 [Fig F2] [Fig F3] –4 , 5A–F , 6A–C , 7A–D , 8

##### Material examined.

**Type series.** **(i)** 1 female juvenile (paratype), PoCL 1.6 mm (OUMNH.ZC.2014.01.016), Marine Science Station area, Aqaba, Jordan, reef wall, 40 m depth, from *Antipathes* sp., leg. Z Ďuriš, 17.06.2008, (fcn-Aq08-25B); **(ii)** 2 ovigerous females (paratypes), PoCL 2.1 mm (RMNH.CRUS.D.56174, GenBank KJ690257), and 2.6 mm (dissected, UO Aq08-34B), Marine Science Station area, Aqaba, Jordan, from antipatharian coral in crevice among concrete blocks of pier, 5 m depth, leg. Z Ďuriš, 16.06.2008 (fcn Aq08-34B); **(iii)** 1 ovig. female **(holotype)**, PoCL 3.3 mm (RMNH.CRUS.D.56175), Marine Science Station pier, Aqaba, Jordan, from antipatharian corals in crevice among concrete blocks of pier, 4 m depth, leg. Z Ďuriš, 20.06.2008 (fcn Aq08-39); **(iv)** 1 ovigerous female (paratype), PoCL 2.4 mm (OUMNH.ZC.2014.01.017), Marine Science Station area, Aqaba, Jordan, reef wall, from *Antipathes* sp., 45 m depth, leg. Z Ďuriš & I Horká, 11.06.2009 (fcn Aq09-8); **(v)** 1 male **(allotype)**, PoCL 1.9 mm (RMNH.CRUS.D.56176), 1 ovigerous female (paratype), PoCL 2.2 (RMNH.CRUS.D.56177, Genbank KJ690256), 1 ovigerous female, PoCL 2.5 mm, 5 males PoCL 1.6–1.9 mm, 1 spm PoCL 1.6 mm (paratypes) (RMNH.CRUS.D.56179), Marine Science Station area, Aqaba, Jordan, rock on sandy bottom ca 15 m out of reef wall, from *Antipathes* sp., 47 m depth, leg. Z Ďuriš & I Horká, 21.06.2009 (fcn Aq09-28B); **(vi)** 1 ovigerous female (paratype), PoCL 3.1 mm (dissected, dried for SEM; UO Aq09-30A), Marine Science Station area, Aqaba, Jordan, reef wall, from *Antipathes* sp., 55 m depth, leg. Z Ďuriš & I Horká, 22.06.2009 (fcn Aq09-30A); **(vii)** 1 ovigerous female PoCl 2.5 mm, 2 females 1.9 mm, 1 male PoCL 1.5 mm (paratypes) (UO Aq09-65), Marine Science Station area, Aqaba, Jordan, sandy slope, antipatharian coral on rock, 50–55 m depth, leg. Z Ďuriš & I Horká, 01.06.2009 (fcn Aq09-65); **(viii)** 1 ovigerous female (paratype), PoCL 2.3 mm (RMNH.CRUS.D.56180), Marine Science Station area, Aqaba, Jordan, from *Antipathes* sp., 55 m depth, leg. Z Ďuriš & I Horká, 05.06.2009 (fcn Aq09-93).

**Non-type material.** **(ix)** 1 female, PoCL 3.3 mm (OUMNH.ZC.2011.05.078), Eilat, Israel, 29°30'07"N, 34°55'05"E, 40 m depth, from *Antipathes* sp., leg. S De Grave & ML Johnson, 04.04.2011 (fcn ISR-092); **(x)** 3 ovig. females (PoCL 2.6, 3.0, 3.2 mm) (OUMNH.ZC.2011.05.079), Eilat, Israel, 29'51"N, 34°55'39"E, 22 m depth, from *Antipathes* sp., leg. S De Grave & ML Johnson, 29°, 05.04.2011 (fcn ISR-112).

##### Diagnosis.

Carapace dorsally trilobate in adults. Rostrum short, not reaching end of eyes; deep and anteriorly serrated with up to 6 small secondary teeth of which upper second or third tooth extending furthest forward; posterior margin smooth and convex; lower margin unarmed, slightly convex. Posterior dorsal lobe on carapace strongly hooked in adults. Posterior propodal spinule of ambulatory pereiopods small and single. Third abdominal segment distinctly angulated in lateral aspect, with dorsally straight outline. Sixth abdominal segment 2.5 times longer that deep. Mesial margin of endopod of male first pleopod with 9 mesial setae. Appendix masculina of second male pleopod longer than appendix interna. Telson with 3–5 pairs of dorsal spines.

##### Description of female holotype.

Carapace (Fig. 1A) smooth, about twice as long as height, with 2 large triangular teeth on dorsal midline. Anterior tooth placed just behind posterior level of orbit, similar to but slightly smaller than rostrum, anteriorly serrated with 4 small teeth; small obtuse epigastric tooth placed posteriorly on base of anterior tooth. Large posterior tooth placed on posterior third of carapace, higher than anterior tooth and strongly hooked with apex pointed forwards, and with anterior and posterior margins convex, smooth, without serration; small acute anteriad directed tooth situated posterodorsally near margin of carapace. Dorsal margin of orbit continuous with short dorsal midrib extending to middle of rostrum near most anteroventrally tooth of rostrum; lower orbital angle broadly rounded, not produced. Antennal tooth well developed, marginal, acute; supraorbital, hepatic and pterygostomial teeth absent; pterygostomial angle subquadrate, obtuse.

Abdomen smooth and compressed, with all pleurae rounded posteriorly. Third segment produced posterodorsally, with straight dorsal outline. Sixth segment twice as long as fifth segment and more than twice as long as maximal depth; posterolateral and posteroventral angles obtusely produced. Abdominal sternites unarmed, sixth sternite with small preanal tubercle.

**Figure 2. F2:**
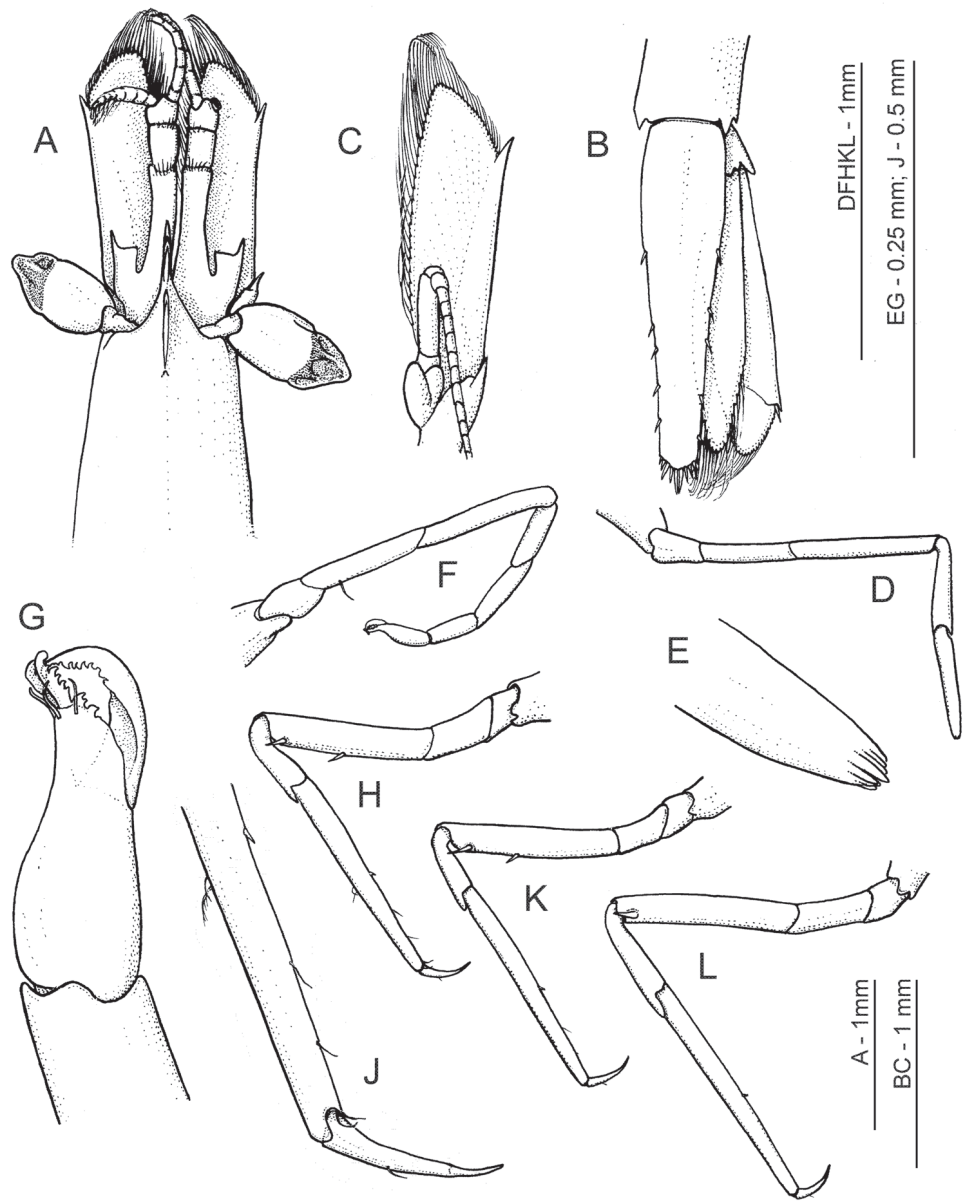
*Anachlorocurtis occidentalis* sp. n., holotype, ovigerous female (RMNH.CRUS.D.56175). **A** anterior carapace, antennae and eyes, dorsal **B** telson and uropod, dorsal **C** antenna, ventral **D** right first pereiopod **E** same, terminal segment **F** right second pereiopod **G** same, chela, ventrolateral aspect **H** third pereiopod, lateral **J** same, dactylus and distal propodus **K** fourth pereiopod **L** fifth pereiopod.

Telson (Fig. 2B) slender, as long as sixth abdominal segment and 4 times longer than maximal width at anterior fourth; with 5 pairs of small (less than 0.05 of telson length), irregularly placed dorsolateral spinules, first pair placed at 0.35 of telson length; the following 4 pairs distributed along distal half of telson. Posterior margin broadly rounded, with 5 pairs of spines, lateral shorter than dorsal spines, remaining spines longer, with submedian pair longest but not reaching 0.1 of telson length.

Eyes (Fig. 5F) long and cylindrical; cornea well pigmented, shorter than stalk, with a distinct pointed apical tubercle; without accessory pigment spot.

Antennular peduncle (Fig. 2A) with basal segment long, more than twice as long as distal 2 segments combined, with several long plumose seta on distal dorsal margin; mesial margin with deep and thin ventral keel along whole segment. Stylocerite reaching about middle of basal segment; outer margin nearly straight, ending in acute spine far overreaching transverse anterior margin; inner distal angle angulate, pointed. Distal 2 segments subequal, short and broad. Upper flagellum short, composed of about 9 segments with 4 basal segments swollen, and with about 6 groups of aesthetascs on distal segments; lower flagellum slightly longer than upper flagellum.

Antenna (Fig. 2C) with basicerite bearing well developed distolateral tooth; carpocerite cylindrical, reaching middle of scale. Scaphocerite distinctly exceeding antennular peduncle, about 3 times as long as broad; outer margin straight, ending in a stout spine, far overreached by angulate distomesial part of lamina. Flagellum slender, long, about half as long as body.

First pereiopod (Fig. 2D–E) slender, not chelate, reaching apex of rostrum; dactylus fully reduced, possibly indicated only as part of terminal spinulation of propodus; propodus about 7.0 times longer than basal depth, slightly tapering to apex; carpus, merus and ischium elongate, unarmed, almost uniformly wide, and about 0.8, 1.3 and 0.8 of propodus length, respectively.

Second pereiopod (Fig. 2F–G) slender, chelate, not reaching distal end of eye; chela small, spatulate, with large gap between fingers, dactylus curved, with distally denticulate lateral cutting edge, fixed finger short, stout, with several irregular distal teeth on cutting margin, with pair of large denticulate spines with hooked apices on apex, and with several simple setae subterminally; palm slightly longer than dactylus, swollen proximally; carpus about 3 times as long as chela, three-jointed, with length ratios 1.0: 1.7: 1.4 (distal to proximal), proximal segment obliquely articulating intermediate one; merus two-thirds length of carpus; ischium as long as merus.

Third pereiopod (Fig. 2H, J) more robust than first 2 pereiopods, reaching end of basal antennular segment; dactylus slender, 5.0 times longer than basal depth, curved, with a distinct unguis; propodus 3.5 times as long as dactylus and about 10 times longer than basal width, tapering distally, with one distinct spinule in middle of ventral margin, without distoventral spines near articulation with dactylus; carpus about 0.4 of propodus length and slightly deeper distally than propodus, distodorsal end extending over base of propodus as flat expansion; merus about 0.8 of propodus length and about 6.0 times longer than uniform width, with 2 distinct ventrolateral spines, one subterminal and one at midlength of segment; ischium, basis and coxa short, unarmed.

Fourth and fifth pereiopods (Fig. 2K, L) subequal to third pereiopod, fifth slightly longer and more slender than fourth; fourth pereiopod similar to third pereiopod in spination of both propodus and merus, with proximal spine placed more forwards (distal 0.6 of ischium length); fifth pereiopod merus armed with one subterminal spine only.

Endopod of first pleopod reduced, triangular, appendix interna without cincinnuli, with marginal plumose setae.

Uropod (Fig. 2B) with branches slightly shorter than telson; outer margin of exopod almost straight, terminating in short fixed spine and with more mesial movable spine about twice overreaching fixed tooth; diaeresis well developed, unarmed mesially from lateral movable spine.

##### Description of mouthparts

**Figure 3. F3:**
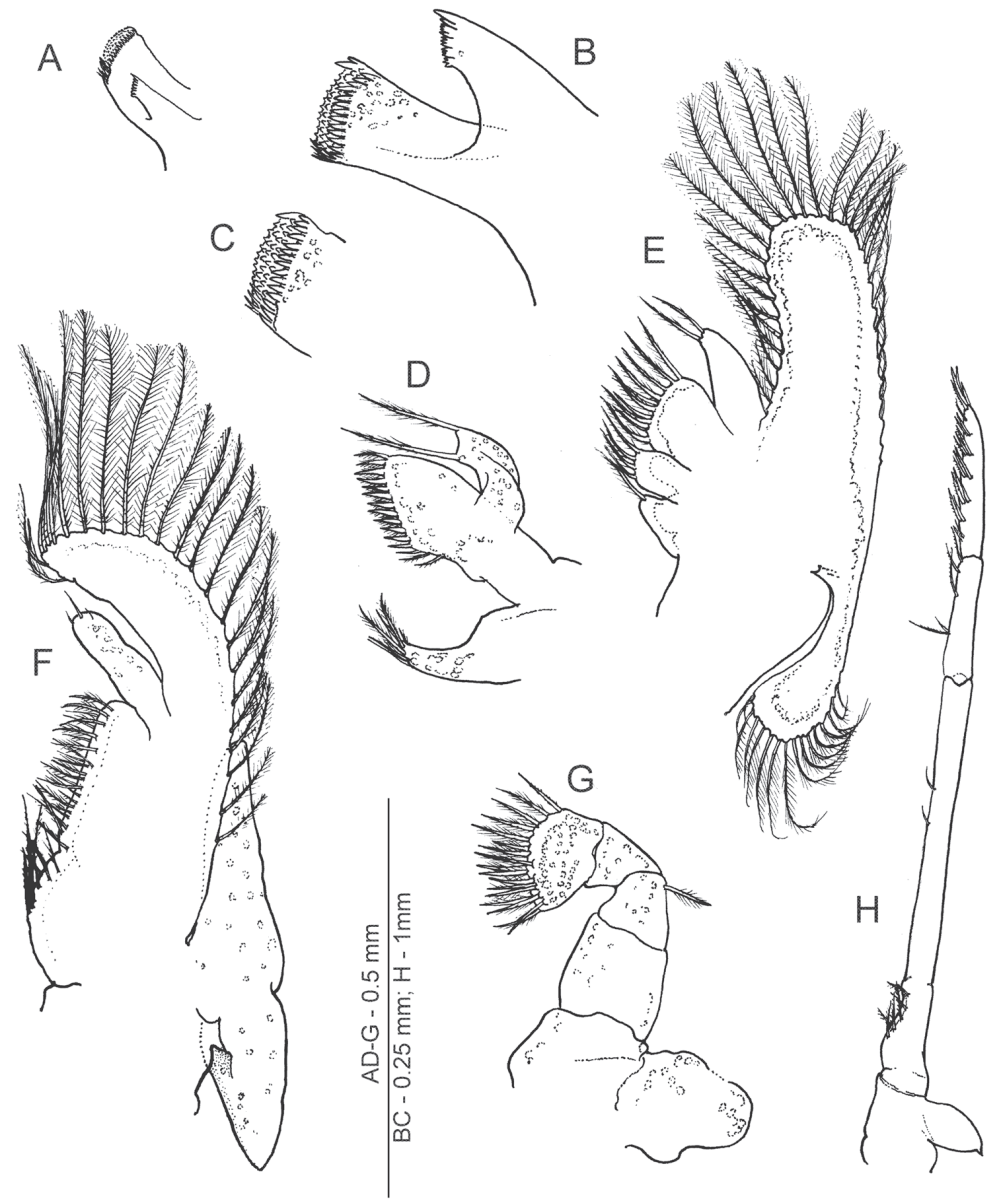
*Anachlorocurtis occidentalis* sp. n., mouthparts (ovigerous female, PoCL 2.6 mm, UO Aq08-34B). **A** mandible, anterior aspect **B** same, molar and incisor processes, anteroventral **C** same, apex of molar process, anteromesial **D** maxillula **E** maxilla **F** first maxilliped **G** second maxilliped **H** third maxilliped.

(paratype – ovigerous female PoCL 2.6 mm; UO Aq08-34B). Mandible (Fig. 3A–C) without palp; molar process with broadly truncate distal end densely covered by sharp tubercles and some slightly larger marginal teeth and subequal stout setae; incisor process basally broad, tapering distally, with obliquely truncate distal end comprised of 4 subquadrate, distally serrate, proximal teeth, and 2 slender simple teeth, terminal one more produced.

Maxillula (Fig. 3D) with palp truncate distally and bearing 2 long pappose setae; broad distal lacinia armed with numerous strong pappose setae; basal lacinia elongate, slender, tapering distally, with about 6 pappose or simple setae terminally.

Maxilla (Fig. 3E) with short palp about 2.5 times longer than broad basally, with 2 pappose setae distally; scaphognathite well developed, anterior lobe produced, almost twice longer than broad basally, posteriorly rounded, marginal setae densely plumose. Distal endite divided into 2 parts with plumose setae marginally; basal endite not divided and with single apical pappose seta.

First maxilliped (Fig. 3F) with elongate palp, more than 3.0 times longer than wide basally, with 3 simple setae distally; exopod composed of caridean lobe with long plumose marginal setae, flagellum absent; endites separated by faint incision, with sparsely distributed pappose setae; epipod broadly triangular, faintly bilobed.

Second maxilliped (Fig. 3G) with dactylar segment completely fused to propodus, mesial margin with more than 15 stout pappose setae; single pappose seta situated distolaterally on merus; ischium broader and longer than carpus; basis and coxa fused, with division faintly indicated; exopod absent; epipod large, broadly ovate.

Third maxilliped (Fig. 3H) slender and long, failing to reach end of basal antennular segment; lateral coxal plate ovate, with small spiniform apex; antepenultimate segment (i.e., merus, ischium and basis fused) with subdivisions feebly indicated, about 12 times longer than uniform width and 1.5 times as long as distal 2 segments combined; setose concavity present on ventral margin of ischium; penultimate segment (i.e., carpus) about 4.0 times longer than wide basally and 0.4 of preceding segment length, with several serrulate setae ventrally; ultimate segment about 1.2 times longer than penultimate segment, furnished with about 8 dorsomesial rows of serrulate setae.

##### Other specimens.

**Figure 4.**
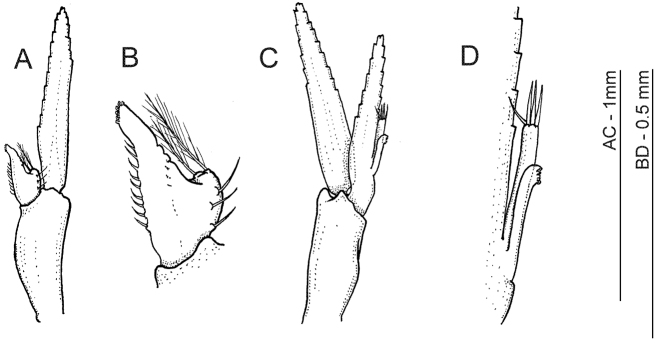
*Anachlorocurtis occidentalis* sp. n., allotype, male (RMNH.CRUS.D.56176). **A** first pleopod **B** same, endopod **C** second pleopod **D** same, mesial margin of endopod with appendices interna and masculina.

**Figure 5.**
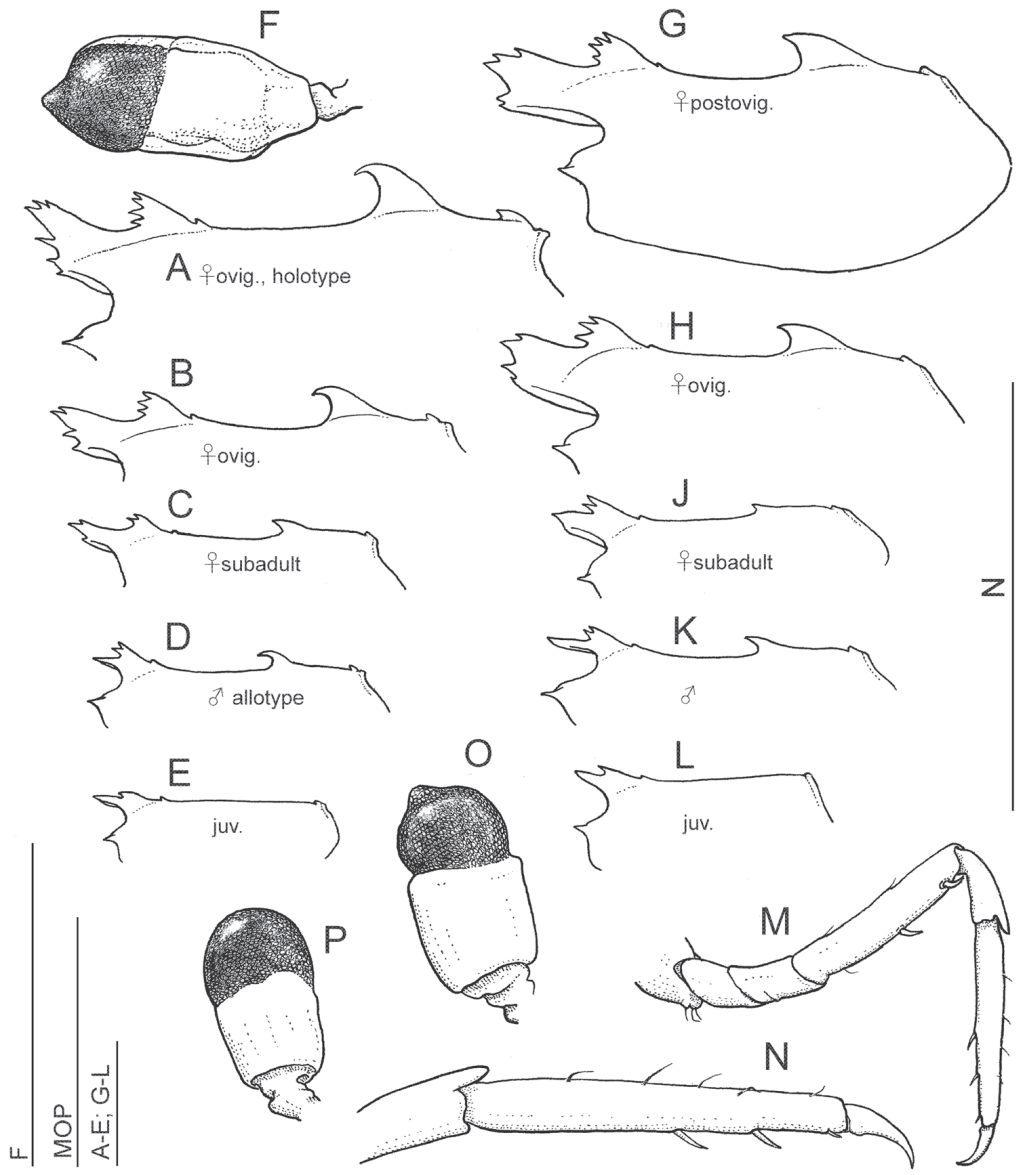
*Anachlorocurtis occidentalis* sp. n., (**A–F** Aqaba, 2009) and *Anachlorocurtis commensalis* Hayashi, 1975 (**G–P** Taiwan, 2009), outline of carapace, lateral aspect (**A–E, G–L**), third pereiopod (**M–N**), and eye (**F, O–P**). **A** holotype, ovigerous female PoCL 3.3 mm (RMNH.CRUS.D.56175) **B** ovigerous female PoCL 2.5 mm (RMNH.CRUS.D.56179) **C** subadult female PoCL 1.9 mm (UO Aq09-65) **D** allotype, male PoCL 1.9 mm (RMNH.CRUS.D.56176) **E** juvenile specimen PoCL 1.6 mm (RMNH.CRUS.D.56179) **F** ovigerous female PoCL 2.5 mm (RMNH.CRUS.D.56179) **G** post-ovigerous female PoCL 2.6 mm (OUMNH.ZC.2010-02-010 )**H** ovigerous female PoCL 2.6 mm **J** subadult female PoCL 1.9 mm **K** male PoCL 2.2 mm **L** juvenile specimen PoCL 1.6 mm (OUMNH.ZC.2010.02.061) **M–O** post-ovigerous female PoCL 2.6 mm (OUMNH.ZC.2010-02-010) **P** male PoCL 2.2 mm. Scale bars equal 1 mm.

Males generally similar to adult females but distinctly smaller and more slender in lateral aspect (see Fig. 1). Allotype male (Figs 1B, 5D) with rostrum simple, styliform, with anteriad directed, bifid, dorsal tooth placed just above level of posterior orbital margin, and with obtuse epigastric tooth more posteriorly; posterior hooked tooth well developed at posterior third of carapace, posterior submarginal tooth small but distinct. Upper antennular flagellum with basal swollen part (corresponding to fused flagella) more elongate than in adult females, with stronger developed aesthetascs. Endopod of first pleopod (Fig. 4A, B) reduced to small setose lobe with bilateral setae and pair of pappose terminal setae, appendix interna far overreaching reduced endopod but falling short of midlength of exopod, elongate triangular, with 9 simple hooked spiniform setae on mesial margin, and with group of about 8 cincinnuli on oblique apex. Endopod of the second pleopod (Fig. 4C) almost as long as exopod, with pair of appendices emerging from proximal third of mesial margin; appendix masculina slender, more than 6.0 times longer than basal width, reaching midlength of endopod; with 4 stout simple setae on apex. Appendix interna reaching 0.75 of appendix masculina length, with about 8 terminal cincinnuli (Fig. 4D).

##### Variation.

The main morphological variation observed within the samples of the new species is in the dorsal armament of the carapace and rostrum (Fig. 5A–E). While in adult and subadult females the carapace is trilobate, comprised of 2 high, anteriorly denticulate, similar “lobes”, i.e., the rostrum and the anterior carapacial lobe, and a large posterior hooked tooth, in males only the postrostral lobe is developed, being small and bidentate, in addition to a short styliform rostrum and the posterior hooked tooth. In juveniles, the posterior hooked tooth is absent, and the anterior ornamentation generally consists of a short simple rostrum with a single posterior dorsal tooth. The rostral formula (number of anterior dentition of the postorbital lobe + same for rostrum) is 3-4 + 2-6 for adult females, 2-3 + 1-3 for subadult females, 2 + 1 (i.e., simple rostrum) for males, and 1 + 1-3 for juveniles.

The small epigastric tooth is placed some distance from the posterior end of the base of the small anterior tooth on the carapace in juveniles, not on the base of the lobe as in adults. The posterior dorsal submarginal tooth, well developed in adult females, is small but distinct in males and subadult females, and may present also in juveniles.

The dorsal telson dentition generally consists of 3 pairs of spinules, with the first pair situated anteriorly to the midlength of the telson. Some females possess 4 pairs of those spinules, e.g., in a subadult female (UO Aq09-30A), or 5 pairs (ovigerous female holotype, RMNH.CRUS.D.56175).

The specimens are consistent in the number of the meral spines on the 3^rd^-5^th^ pereiopods, 2-2-1, respectively. A limited variation is present in the propodal ventral spinulation in the ambulatory pereiopods. In the females examined, the spinulation is 1-1-1, respectively, with the spinules very minute, while in adult males the spinules are stronger, numbering 2-2-1/2, respectively.

##### Colour in life

**Figure 6.**
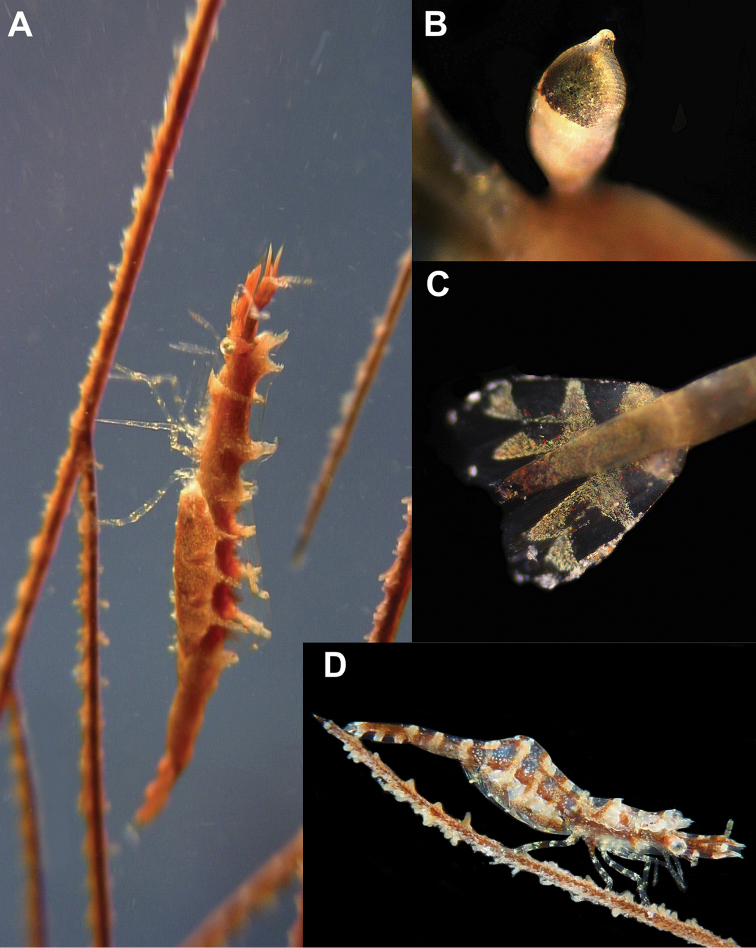
Colour patterns. *Anachlorocurtis occidentalis* sp. n. **A** ovigerous female holotype (RMNH.CRUS.D.56175) on antipatharian host **B** detail of the eye with produced apical tubercle **C** colour patterns of the tail fan. *Anachlorocurtis commensalis* Hayashi, 1975 **D** ovigerous female (UO Tw12-79).

(Fig. 6A–C). Cryptic, mimicking antipatharian host, ventral part of body reddish brown (similar to axial coral branch pattern), dorsal part of body transparent with yellowish to light reddish stripes emerging from internal axis of body (similar to host polyps), with narrow lighter band across carapace; antennulae light brown, scaphocerites with 2 light brown patches (distal and proximal), connected by narrow medial stripe; abdomen with irregular narrow light bands across each segment; tail fan (Fig. 6C) with 3 wide light brown transverse bands across uropods, with colour merged along mesial margin of endopod. Ovigerous females with greyish-brown finely marbled pattern on abdominal pleurae covering egg mass.

**Figure 7.**
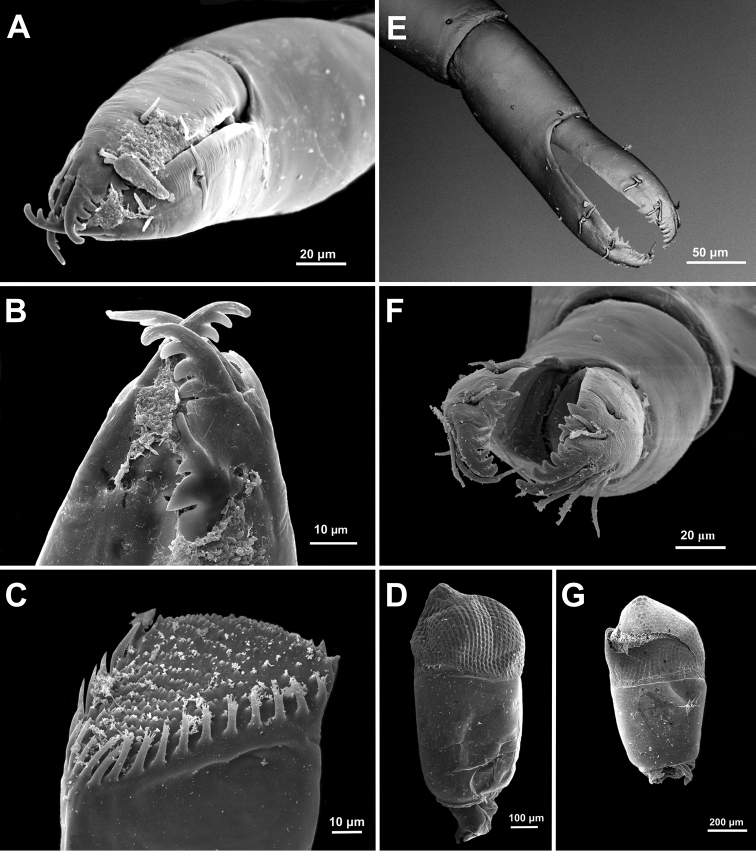
*Anachlorocurtis occidentalis* sp. n., ovigerous female paratype, PoCL 3.1 mm (UO Aq09-30A). **A** second pereiopod chela **B** same, tips of fingers **C** mandible, molar process, antero-mesial aspect (from side of incisor process) **D** eye. *Anachlorocurtis commensalis* Hayashi, 1975, ovigerous female PoCL 2.5 mm (UO Tw12-79) **E** second pereiopod chela **F** same, tips of fingers **G** eye.

##### Measurements.

Holotype ovigerous female: PoCL 3.3 mm, RL 0.8 mm, TL about 12 mm, eggs (without eye-spots) diameters 0.58 × 0.42 mm. Allotype male: PoCL 2.0 mm, RL 0.23 mm, TL 8.7 mm. Paratypes: males (5 spms) PoCL 1.6–1.8 mm, TL 7.1–8.2 mm; ovigerous females (8 spms) PoCL 2.2–3.1 mm, TL 8.7–11.5 mm; subadult females (2 spms) PoCL 1.9 mm; juveniles (2 spms) PoCL 1.5-1.6 mm; eggs without eyespots diameters (7 spms) 0.44–0.56 × 0.36–0.40 mm, eggs with eyespots (1 spm) 0.62 × 0.48 mm.

##### Host and habitat.

Specimens were caught on black corals (*Antipathes* sp.), found in deeper waters along the reef wall and on a boat wreck at 22–55 m, but also in dark crevices in shallow waters, only 4–5 m deep.

##### Associated fauna.

The specimens of the present new species were collected from their hosts together with specimens of the pontoniine shrimps, *Manipontonia psamathe* (De Man, 1902) and *Periclimenes* cf. *lepidus* Bruce, 1978, both not previously reported from the Red Sea.

##### Etymology.

The specific name is from the Latin *occidentalis*, western, reflexing the geographic range of the new species in the westernmost region of the Indo-West Pacific area, the Red Sea, as opposed to the East Asian distribution of the type species of the genus.

##### Distribution.

Currently only known from the type locality, Gulf of Aqaba, in the north-eastern Red Sea.

#### Anachlorocurtis commensalis

Hayashi, 1975

http://species-id.net/wiki/Anachlorocurtis_commensalis

**Figure 8.**
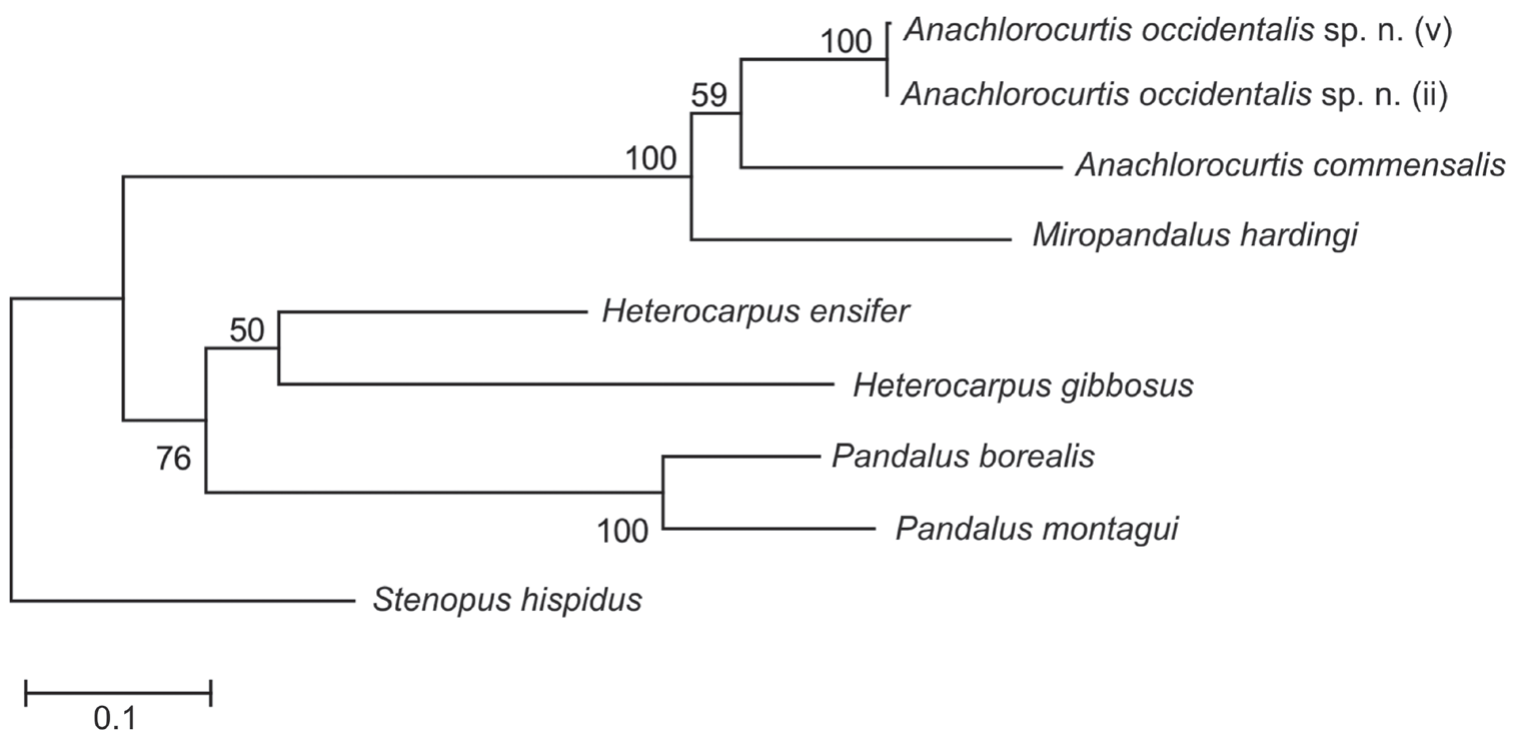
Phylogenetic tree obtained by maximum likelihood analysis (GTR+G+I substitution model) of partial sequence of COI (658 bp).

Figs 5G–P , 6D , 7E–G , 8

Anachlorocurtis commensalis Hayashi 1975: 172–182, figs 1–3.; Hayashi 2007: 147–150, figs 538–539.; Kawamoto and Okuno 2003: 64, colour photo.; Minemizu 2013: 129, colour photo.

##### Material examined.

**(i)** 8 spms (1 ovigerous female PoCL 2.6 mm, 1 female subadult PoCL 2 mm, 3 males PoCL 1.8–2.1 mm, 3 juveniles PoCL 1.6–1.8 mm) (OUMNH.ZC.2010.02.061), Gudanshr reef, Namwan Bay, Pingtung County, Taiwan, 21°56.531'N, 120°45.546'E, from bushy black coral, 10 m depth, leg. S De Grave, 07.12.2009 (fcn TAI-244); **(ii)** 10 spms (4 females ovigerous PoCL 2.5–2.6 mm, 1 female post ovigerous PoCL 2.6 mm, 1 female PoCL 2.3 mm, 1 female subadult PoCL 1.9 mm, 3 males PoCL 1.9–2.2 mm) (OUMNH.ZC.2010-02-010), Gudanshr reef, Namwan Bay, Pingtung County, Taiwan, 21°56.531'N, 120°45.546'E, from *Cirrhipathes* sp., 27 m depth, leg. S De Grave, 12.12.2009 (fcn TAI-327); **(iii)** 1 juvenile PoCL 1.8 mm (RMNH.CRUS.D.56181), Gudanshr reef, Namwan Bay, Pingtung County, Taiwan, from antipatharian on overhanging rock wall, 10.9 m depth, leg. Z Ďuriš, 11.11.2011 (fcn Tw11-11); **(iv)** 1 juvenile (damaged) PoCL 1.8 mm (OU Tw11-20B), Gudanshr reef, Namwan Bay, Pingtung County, Taiwan, upper side of rocky reef, from yellow branching antipatharian, 18.6 m depth, leg. Z Ďuriš, 03.11.2011 (fcn Tw11-20B); **(v)** 1 ovigerous female PoCL 2.3 (RMNH.CRUS.D.56182, Genbank KJ690258), 1 ovigerous female, PoCL 2.4 mm, 1 male PoCL 2.1 mm (NTOU M01305), Gudanshr reef, Namwan Bay, Pingtung County, Taiwan, upper side of reef, from dense bushy antipatharian, 22.1 m depth, leg. Z Ďuriš, 03.11.2011 (fcn Tw11-21); **(vi)** 1 ovigerous female PoCL 2.5 mm (dissected, dried for SEM; UO Tw12-79), Gudanshr reef, Namwan Bay, Pingtung County, Taiwan, reef slope, from antipatharian, 25.3 m depth, leg. Z Ďuriš & I Horká, 12.09.2012 (fcn Tw12-79).

##### Remarks.

The specimens examined agree well with the original description by Hayashi (1975). As in *Anachlorocurtis occidentalis* sp. n., the specimens examined of *Anachlorocurtis commensalis* vary in the dorsal armament of the carapace and rostrum (Fig. 5G–L). In adult and subadult females the carapace with the rostrum is triple lobate, in contrast in males only the anterior, bidentate, lobe is developed, in addition to the posterior hooked tooth and a short styliform rostrum. In juveniles the posterior hooked is absent and the anterior ornamentation generally consists of a short simple rostrum with a single posterior dorsal tooth. The posterior dorsal submarginal tooth is small in adults and subadults, and is absent in juveniles. The rostral formula (see above) is 2-4 + 4-5 for adult females, 2-3 + 1-2 for subadult females, 2 + 1 (i.e. simple rostrum) for males, and 1-2 + 1 for juveniles. Cornea in males is almost globular, with the apical tubercle indistinct, in females the tubercle is more produced but not as developed as *Anachlorocurtis occidentalis* sp. n.

##### Colour in life.

Generally similar to that of *Anachlorocurtis occidentalis* sp. n., but the light transverse bands are greyish, more wide and distinctive in *Anachlorocurtis commensalis* (Fig. 6D); also see colour photos in Minemizu (1996: 8; 2000: 113; 2013: 129), Kato and Okuno (2000: 58) and Kawamoto and Okuno (2003: 64).

##### Distribution.

Kii Peninsula (type locality), Izu-Oshima and Hachijyo-jima, Izu Islands, and Kume-jima, Ryukyus, Japan (Minemizu 1996, 2000, 2013, Kato and Okuno 2000), Kawamoto and Okuno (2003); South (Namwan) Bay, Taiwan (present report).

### Molecular data

Molecular analysis (Maximum Likelihood, Fig. 8) supports a sister position of *Anachlorocurtis occidentalis* sp. n. (Red Sea) to *Anachlorocurtis commensalis* (Taiwan). Both species form a monophyletic clade with *Miropandalus hardingi*. The maximum Kimura 2-parameter distance between *Anachlorocurtis occidentalis* sp. n. and *Anachlorocurtis commensalis* reached 15.4% (for more details see Table 2).

**Table 2.** Kimura’s 2-parameter distances of COI gene sequences among species studied.

	*Anachlorocurtis occidentalis* sp. n.	*Anachlorocurtis occidentalis* sp. n.	*Anachlorocurtis commensalis*	*Miropandalus hardingi*	*Heterocarpus ensifer*	*Heterocarpus gibbosus*	*Pandalus borealis*	*Pandalus montagui*	*Stenopus hispidus*
*Anachlorocurtis occidentalis* sp. n. (RMNH.CRUS.D.56177)	–								
*Anachlorocurtis occidentalis* sp. n. (RMNH.CRUS.D.56174)	0.002	–							
*Anachlorocurtis commensalis*	0.154	0.152	–						
*Miropandalus hardingi*	0.156	0.154	0.192	–					
*Heterocarpus ensifer*	0.254	0.252	0.267	0.268	–				
*Heterocarpus gibbosus*	0.300	0.297	0.295	0.272	0.211	–			
*Pandalus borealis*	0.278	0.275	0.286	0.293	0.221	0.236	–		
*Pandalus montagui*	0.268	0.266	0.276	0.270	0.220	0.246	0.123	–	
*Stenopus hispidus*	0.253	0.251	0.237	0.248	0.219	0.226	0.251	0.230	–

## Discussion

*Anachlorocurtis occidentalis* sp. n. is morphologically closely related to the only other species in the genus, *Anachlorocurtis commensalis*. Both species have a small, slender body and ambulatory legs. The third to fifth pereiopods have the propodi about 10 times longer than deep basally *Anachlorocurtis occidentalis*, whilst 8 times longer in *Anachlorocurtis commensalis*, and the dactyli are 5.5 times longer than deep basally in the new species, and 3 times longer than deep in *Anachlorocurtis commensalis*. The new species also differs from *Anachlorocurtis commensalis* by the posterior dorsal lobe on the carapace being strongly hooked in adults (vs. feebly hooked), and more developed posterior submarginal tooth dorsally on the carapace in adults; by a more elongate apical tubercle on cornea; by a small and single posterior propodal spinule on the 3^rd^ - 5^th^ pereiopods (vs. stronger, with their number 2-2-2, respectively, in *Anachlorocurtis commensalis*); by the distinctly angulated third abdominal segment in lateral aspect, with dorsally straight outline (vs. rounded); by the more elongated sixth abdominal segment (2.5 times longer that deep in *Anachlorocurtis occidentalis* vs. 2.0 in *Anachlorocurtis commensalis*); by the greater number of setae on the mesial margin of the endopod of the first pleopod of the male (9 versus 3, respectively); by the length of the appendix masculina of the second male pleopod (longer than the appendix interna versus shorter), and possibly a higher number of dorsal telson spines (3–5 pairs in *Anachlorocurtis occidentalis*, versus 3 pairs in *Anachlorocurtis commensalis*).

The morphological distinction between the two species is further supported by small differences in their colour pattern, but more importantly by their COI divergence being 15.2–15.4%.

## Supplementary Material

XML Treatment for Anachlorocurtis

XML Treatment for Anachlorocurtis occidentalis

XML Treatment for Anachlorocurtis commensalis
